# The Dual Burden: Exploring Cardiovascular Complications in Chronic Kidney Disease

**DOI:** 10.3390/biom14111393

**Published:** 2024-10-31

**Authors:** Alfredo Caturano, Raffaele Galiero, Maria Rocco, Giuseppina Tagliaferri, Alessia Piacevole, Davide Nilo, Giovanni Di Lorenzo, Celestino Sardu, Vincenzo Russo, Erica Vetrano, Marcellino Monda, Raffaele Marfella, Luca Rinaldi, Ferdinando Carlo Sasso

**Affiliations:** 1 Department of Advanced Medical and Surgical Sciences, University of Campania Luigi Vanvitelli, 80138 Naples, Italy; alfredo.caturano@unicampania.it (A.C.); raffaele.galiero@unicampania.it (R.G.); rocco.maria92@gmail.com (M.R.); giuseppina.tagliaferri@gmail.com (G.T.); alessia0694@hotmail.it (A.P.); nilodavide@gmail.com (D.N.); giuann86@gmail.com (G.D.L.); celestino.sardu@unicampania.it (C.S.); erica.vetrano@unicampania.it (E.V.); raffaele.marfella@unicampania.it (R.M.); 2 Department of Experimental Medicine, University of Campania Luigi Vanvitelli, 80138 Naples, Italy; marcellino.monda@unicampania.it; 3 Division of Cardiology, Department of Medical Translational Sciences, University of Campania Luigi Vanvitelli, 80138 Naples, Italy; v.p.russo@libero.it; 4 Department of Biology, College of Science and Technology, Sbarro Institute for Cancer Research and Molecular Medicine, Temple University, Philadelphia, PA 19122, USA; 5 Department of Medicine and Health Sciences “Vincenzo Tiberio”, Università degli Studi del Molise, 86100 Campobasso, Italy

**Keywords:** chronic kidney disease, cardiovascular risk, pharmacological management, lifestyle modifications, renal function, novel treatment

## Abstract

Chronic kidney disease (CKD) represents a significant global health challenge, affecting millions of individuals and leading to substantial morbidity and mortality. This review aims to explore the epidemiology, cardiovascular complications, and management strategies associated with CKD, emphasizing the importance of preventing cardiovascular disease and early intervention. CKD is primarily driven by conditions such as diabetes mellitus, hypertension, and cardiovascular diseases, which often coexist and exacerbate renal impairment. Effective management requires a multifaceted approach, including lifestyle modifications, pharmacological interventions, and regular monitoring. Dietary changes, such as sodium restriction and a controlled intake of phosphorus and potassium, play a vital role in preserving renal function. Pharmacological therapies, particularly angiotensin-converting enzyme (ACE) inhibitors, angiotensin receptor blockers (ARBs), and emerging agents like SGLT2 inhibitors, have shown efficacy in slowing disease progression and improving patient outcomes. Furthermore, patients undergoing dialysis face increased cardiovascular risk, necessitating comprehensive management strategies to address both renal and cardiac health. As the landscape of CKD treatment evolves, ongoing research into novel therapeutic options and personalized medical approaches are essential. This review underscores the urgent need for awareness, education, and effective preventive measures to mitigate the burden of CKD and enhance the quality of life for affected individuals.

## 1. Introduction

Chronic kidney disease (CKD) is a long-term condition characterized by a gradual decline in kidney function, often progressing to end-stage renal disease (ESRD), where dialysis or a kidney transplant becomes necessary [1]. CKD is commonly categorized into six categories, determined by the glomerular filtration rate (GFR) and the presence of kidney damage (albuminuria categories (A1–A3)). The categories range from G 1 (normal or high GFR with kidney damage) to G 5 (GFR of less than 15 mL/min/1.73 m^2^, indicative of kidney failure) [2]. This classification helps in assessing the severity of the disease and guiding treatment decisions [2].

The global prevalence of CKD continues to pose a significant public health challenge, affecting an estimated 9.5% of the global population, with rates as high as 12.8% in certain regions such as Eastern and Central Europe [3]. Recent studies report that the prevalence of CKD among adults worldwide is approximately 13.4% for stages 1–5 and 10.6% for stages 3–5 [4]. In low- and middle-income countries, CKD prevalence can exceed 20%, exacerbating health disparities [4]. According to the Global Burden of Disease Study, CKD ranked as the 12th leading cause of death worldwide in 2017, and the mortality rate increased by 41.5% between 1990 and 2017 [5]. This alarming trend is largely driven by the rising incidence of major risk factors, including diabetes, hypertension, obesity, and aging populations [5]. Its progression can lead to ESRD, requiring dialysis or transplantation, which imposes substantial healthcare costs and affects patients’ quality of life [6]. Thus, understanding the epidemiology of CKD and its associated risk factors is crucial for implementing effective preventive strategies [7]. The primary risk factors for CKD include diabetes mellitus, hypertension, and cardiovascular diseases [8,9,10]. These conditions often coexist and contribute to the deterioration of renal function through various mechanisms such as hyperfiltration, inflammation, and oxidative stress [11,12]. Early detection of these risk factors through routine screenings can facilitate timely interventions, potentially slowing the progression of CKD [13]. In addition, educational campaigns targeting high-risk populations can raise awareness about the importance of lifestyle changes, adherence to medication, and regular health check-ups [14]. The presence of CKD significantly increases the risk of developing cardiovascular diseases (CVDs), which are the leading cause of morbidity and mortality in this patient population [8,15]. Individuals with CKD are more likely to suffer from conditions such as ischemic heart disease, heart failure, arrhythmias, and sudden cardiac death [16]. This heightened cardiovascular risk is attributed to a combination of traditional risk factors (e.g., hypertension, hyperlipidemia, and diabetes) and non-traditional risk factors that are specific to CKD, such as altered calcium–phosphate metabolism, chronic inflammation, oxidative stress, and anemia [17,18,19,20,21,22].

CKD induces structural and functional changes in the cardiovascular system, including left ventricular (LV) hypertrophy, arterial stiffness, and atherosclerosis [23,24]. These changes are exacerbated by CKD-related factors like uremic toxins and fluid overload, which further strain the heart and vascular system. Additionally, CKD patients often present with a unique lipid profile—characterized by high triglycerides and low high-density lipoprotein (HDL) cholesterol—which contributes to atherosclerosis and cardiovascular risk [25,26,27,28]. Conversely, CVD can worsen kidney function. For instance, heart failure can lead to renal congestion and reduced renal perfusion, accelerating kidney damage. This bidirectional interaction is a hallmark of what is often referred to as the “cardio-renal syndrome”, a complex interplay where dysfunction in one organ system (cardiac or renal) can cause or exacerbate dysfunction in the other [29,30,31]. This review aims to explore the intricate and often underappreciated relationship between CKD and CVD. It will provide a comprehensive overview of the pathophysiological mechanisms that link these conditions, including the roles of inflammation, oxidative stress, mineral bone disorder, and dyslipidemia. Additionally, this review will discuss the clinical implications of this dual burden, such as the challenges in diagnosing and managing CVD in CKD patients, and the therapeutic implications.

## 2. Pathophysiological Interplay Between CKD and CVD

**Figure 1:**
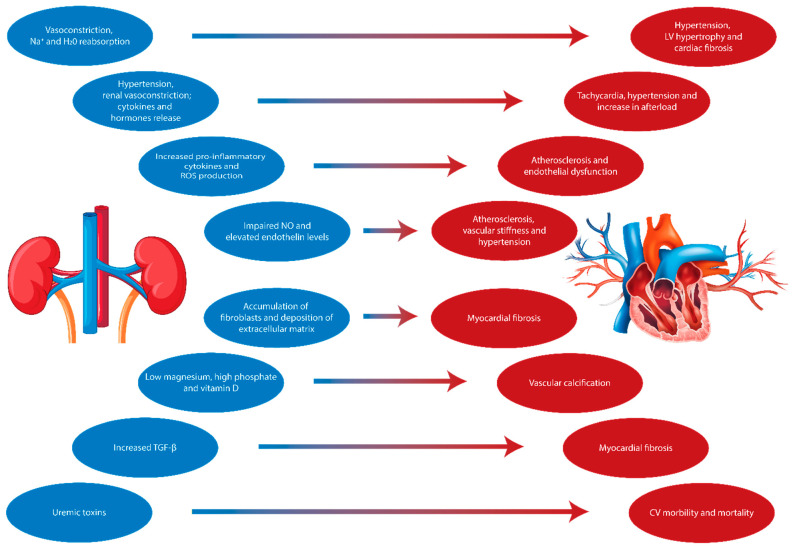
This diagram illustrates the complex mechanisms linking kidney dysfunction to cardiovascular complications. Impaired renal function triggers various pathophysiological processes, including vasoconstriction, sodium (Na⁺) and water (H_2_O) reabsorption, increased release of pro-inflammatory cytokines and reactive oxygen species production (ROS), and impaired nitric oxide (NO) production. These processes contribute to hypertension, vascular stiffness, atherosclerosis, myocardial fibrosis, and left ventricular (LV) hypertrophy. The accumulation of fibroblasts and extracellular matrix deposition, along with increased levels of transforming growth factor beta (TGF-β) and uremic toxins, exacerbate cardiovascular (CV) morbidity and mortality through myocardial fibrosis, vascular calcification, and endothelial dysfunction.

CKD is defined by abnormalities in kidney function or structure, identified by the presence of albuminuria or a reduction in the glomerular filtration rate (eGFR < 60 mL/min/1.73 m^2^) that lasts for more than 3 months [2,32]. CKD substantially increases the risk of adverse outcomes, with CVD being the leading cause of death in this population [33]. Cardiovascular events are already more frequent in early CKD compared to the general population, and this risk escalates as kidney function declines [34]. In addition to traditional risk factors, CKD contributes to systemic conditions that exacerbate vascular damage (Figure 1) [35].

The kidneys, in response to injury, release hormones, enzymes, and cytokines that contribute to a chronic inflammatory state, leading to vascular and myocardial remodeling, atherosclerosis, vascular calcification, and myocardial fibrosis [36,37]. CKD thus accelerates cardiovascular aging [38]. Hypertension, diabetes with its several microvascular complications [10,39,40] obesity, and dyslipidemia are major CVD risk factors, with hypertension being a hallmark of CKD [41,42]. Reduced nephron function leads to sodium retention and fluid overload, which stimulates the renin–angiotensin–aldosterone system (RAAS), promoting vasoconstriction, sodium reabsorption, and further hypertension [41]. Other contributors to elevated blood pressure include vascular stiffness and calcification, both of which are markedly accelerated in CKD [42]. Vascular stiffness, caused by impaired nitric oxide (NO) production and elevated endothelin levels, triggers endothelial dysfunction and promotes inflammation and oxidative stress [43,44,45]. The calcification of central arteries increases the pulse wave velocity and cardiac afterload, leading to LV hypertrophy and reduced coronary perfusion, increasing the risk of heart failure [46,47,48,49]. Uremic calcific arteriolopathy, or calciphylaxis, although uncommon, is the most severe form of vascular calcification in CKD, causing skin necrosis and a high mortality rate [50,51].

Electrolyte imbalances, particularly dysmagnesemia, are common in CKD. Hypomagnesemia is often observed among non-dialysis CKD patients, and some authors assess that tubular dysfunction and interstitial fibrosis seem to play a key role in the development of this condition. Hypomagnesemia would impair tubular magnesium reabsorption, which in turn seems to be associated with CKD progression [52]. On the other hand, magnesium inhibits vascular calcification and prevents the formation of hydroxyapatite crystals, potentially slowing the progression of calcification in advanced CKD [53,54,55,56]. Hemodialysis subjects who show mild hypermagnesemia seem to be associated with the lowest mortality rate [52].

CKD is a systemic inflammatory condition with multiple contributing factors, including oxidative stress, intestinal dysbiosis, metabolic acidosis, and reduced renal clearance of cytokines [57,58,59,60]. Pro-inflammatory cytokines increase resting energy expenditure and suppress anabolic hormones such as growth hormone and insulin-like growth factor 1 [61,62]. The causal role of inflammation in CVD among CKD patients was highlighted in secondary analyses from the CANTOS trial, which showed that canakinumab, an IL-1β monoclonal antibody, reduced major adverse cardiovascular events in CKD patients with prior myocardial infarction and persistently elevated C-reactive protein levels [63,64]. Oxidative stress is prevalent in CKD and correlates with elevated inflammatory markers [65,66]. It results from impaired antioxidant defenses and an increased production of reactive oxygen species (ROS) [67,68]. In CKD models, superoxide dismutase activity is reduced, while NADPH oxidase expression is elevated [69]. Nrf2, a key regulator of antioxidant gene expression, may be a potential target for mitigating oxidative stress in CKD, although further research is needed [70].

Uremia, a complication of CKD, is characterized by the accumulation of solutes normally excreted by healthy kidneys [71]. The European Uremic Toxin Work Group periodically updates a list of these compounds, classifying them based on molecular weight, dialytic clearance, and binding properties [72]. Uremic toxins contribute to CKD progression and comorbidities such as CVD [73]. Notable toxins, including asymmetric dimethylarginine (ADMA), beta-2 microglobulin, indoxyl sulfate, and p-cresyl sulfate, are linked to endothelial dysfunction and vascular damage [73,74,75]. For example, indoxyl sulfate (IS) has harmful effects on both cardiac and vascular cells. In vitro studies show that IS increases ROS production, reduces NO availability, and upregulates adhesion molecules like E-selectin and ICAM-1, promoting inflammation and leukocyte–endothelial interactions [76,77,78,79]. Clinically, IS has been associated with vascular calcification, arterial stiffness, and heart failure in patients with ESRD [76]. ADMA, by inhibiting endothelial nitric oxide synthase (eNOS), contributes to endothelial dysfunction and renal fibrosis [74]. It also induces ICAM-1 and VCAM-1 expression via NF-κB activation, enhancing immune responses in atherosclerosis [80]. Although research on uremic toxins has expanded, they are not the sole drivers of CKD and CVD progression. A combined approach targeting uremic toxins and other contributing factors may be essential for effective treatment [74,81].

Anemia is a common complication of CKD, largely driven by reduced erythropoietin production and iron deficiency, which worsens as kidney function declines [82]. This anemia contributes to increased cardiovascular risk by exacerbating LV hypertrophy, promoting myocardial ischemia, and reducing oxygen delivery to tissues, all of which heighten the risk of heart failure and other cardiovascular events [83,84]. In addition, mineral metabolism disorders, including disturbances in calcium, phosphate, and parathyroid hormone (PTH) levels, play a pivotal role in vascular calcification, a hallmark of cardiovascular disease in CKD patients [85]. Hyperphosphatemia, secondary hyperparathyroidism, and hypocalcemia are common in advanced CKD and lead to the deposition of calcium–phosphate complexes in the vasculature, promoting vascular stiffness and calcification [86]. Elevated PTH levels also contribute to bone-mineral disorders and cardiovascular complications, as they stimulate osteoclast activity and increase calcium release from bones, further enhancing vascular calcification [87]. The dysregulation of mineral metabolism, coupled with anemia, significantly contributes to the heightened cardiovascular morbidity and mortality seen in CKD patients [88,89].

## 3. Diagnostic Challenges and Considerations

### 3.1. Limitations of Traditional Cardiovascular Risk Assessment of CKD

The diagnosis and management of cardiovascular complications in patients with CKD present significant challenges, particularly due to the limitations of traditional cardiovascular risk assessment tools [8]. Conventional risk calculators, such as the Framingham Risk Score [90] and the ASCVD Risk Calculator [91], were developed using data from large population studies that predominantly included individuals without CKD. These tools rely heavily on traditional cardiovascular risk factors like age, gender, blood pressure, cholesterol levels, and smoking status [92]. While these models are effective in the general population, they do not adequately capture the multifactorial nature of cardiovascular risk in CKD patients [93].

CKD is associated with several non-traditional risk factors that are insufficiently addressed by standard risk models [94]. For example, patients with CKD frequently experience chronic inflammation, oxidative stress, disrupted mineral metabolism (notably involving calcium and phosphate), anemia, and the accumulation of uremic toxins, all of which significantly elevate cardiovascular risk [95,96]. These factors are intricately linked to the progression of both kidney and heart disease, yet they are not included in most traditional cardiovascular risk calculators [97]. As a result, these tools often underestimate cardiovascular risk in CKD patients, which can lead to inadequate risk stratification and delayed or suboptimal therapeutic interventions [83]. Furthermore, CKD-specific cardiovascular complications, such as LV hypertrophy, arterial stiffness, and accelerated atherosclerosis, are not adequately accounted for by traditional risk models [98]. Traditional risk calculators, which assess cardiovascular risk in a relatively linear and isolated manner, are ill-equipped to handle this complex, bidirectional interaction [99,100]. The inaccuracies of cardiovascular risk estimation in CKD patients highlight the urgent need for more tailored risk assessment tools that incorporate both traditional and non-traditional CKD-specific risk factors. These tools should consider the unique pathophysiological mechanisms in CKD, including the systemic effects of uremic toxins, the impact of chronic inflammation, and the altered lipid profiles often observed in this population. By developing and implementing CKD-specific risk assessment models, clinicians can more accurately and comprehensively evaluate cardiovascular risk, allowing for earlier and more appropriate interventions. This approach is crucial for improving cardiovascular outcomes in CKD patients, who face significantly higher risks of morbidity and mortality from CVD.

### 3.2. Advanced Diagnostic Tools: Role of Biomarkers and Imaging Modalities in Assessing Cardiovascular Risk

As previously mentioned, there is a clear need for tools that can accurately evaluate cardiovascular risk in patients with CKD. Several commonly used biomarkers in clinical practice can assist in this regard. For example, biomarkers such as troponins, brain natriuretic peptide (BNP), and N-terminal pro-BNP (NT–pro-BNP) are well-established for diagnosing acute myocardial infarction (AMI) and congestive heart failure (CHF) exacerbations [99,100]. However, they have also been correlated with an increased left ventricular mass index (LVMI) and higher mortality in CKD patients [101]. It is crucial to note that in more than 80% of asymptomatic individuals with advanced CKD, these biomarkers are chronically elevated, limiting their use in the same way they are applied to individuals without CKD [93].

BNP and NT–pro-BNP are produced by cardiomyocytes in response to myocardial stress and stretching, making them useful markers in conditions such as volume overload, LV hypertrophy, and hypertension [102]. Both peptides are primarily cleared through the kidneys, with NT–pro-BNP being more dependent on renal clearance than BNP, which is also degraded systemically [103]. These peptides show a positive correlation with serum creatinine levels, heart rates, the left ventricular end-diastolic volume (LVEDV), and the left ventricular end-systolic volume (LVESV) and a negative correlation with body mass index (BMI), the estimated glomerular filtration rate (eGFR), and left ventricular ejection fractions (LVEFs) [104,105,106]. Furthermore, NT–pro-BNP, in particular, has been linked to composite outcomes including myocardial infarction, CHF, stroke, and cardiovascular death in CKD patients [101].

Troponins, specifically cardiac isomers, are intracellular proteins released in response to cellular damage. In non-acute settings, elevated troponin levels serve as a strong predictor of future cardiovascular risk [107]. Among patients newly diagnosed with CKD, elevated baseline troponin values have been associated with heart failure, atrial fibrillation, and cardiovascular mortality [108,109,110]. However, determining clear troponin cut-offs for diagnosing myocardial infarction in CKD patients remains a critical area for future research.

Despite these challenges, NT–pro-BNP and troponins can still be effectively used as cardiovascular biomarkers in CKD patients, provided that clinicians apply a nuanced and observant clinical approach [111,112].

## 4. Therapeutic Approaches and Management Strategies

### 4.1. Lifestyle Modifications and Non-Pharmacological Interventions

CKD was once considered an irreversible condition, where therapeutic strategies merely aimed to delay progression to dialysis [113]. However, recent advancements have shifted this perception, as new therapeutic options now offer substantial evidence of halting or even reversing CKD progression [114]. Before implementing pharmacological therapies, there are essential lifestyle modifications that must be integrated into CKD management. These non-pharmacological interventions focus on healthy daily habits, playing a pivotal role in improving patient outcomes [115]. Key lifestyle modifications include dietary adjustments, regular physical activity, and smoking cessation. These behavioral changes are associated with a notable reduction in recurrent cardiovascular events in both patients with a history of acute coronary syndrome and those with renal failure [116]. In terms of dietary changes, sodium intake plays a critical role. A daily sodium intake of 2 g is associated with better blood pressure control, slowing the progression of kidney disease, and reducing the risk of cardiovascular events [117]. Alcohol and soft drinks, even in small amounts, should be avoided due to their negative impact on cardiovascular and renal health [118,119], whereas moderate coffee consumption does not appear to pose similar risks [120]. Beyond sodium restriction, dietary patterns such as the Mediterranean diet and plant-based diets have demonstrated significant benefits for individuals with CKD [121]. The Mediterranean diet, rich in fruits, vegetables, whole grains, olive oil, and lean proteins like fish and legumes, is associated with reduced inflammation, improved cardiovascular outcomes, and better kidney function preservation [122]. Studies have shown that adherence to the Mediterranean diet leads to improved insulin sensitivity, better lipid profiles, and reduced oxidative stress, all of which are critical in slowing CKD progression and reducing cardiovascular risk in this population [123,124]. Additionally, plant-based diets, which emphasize whole plant foods while minimizing animal products, have been shown to lower blood pressure, improve glycemic control, and reduce proteinuria [125]. These diets are also linked to a lower risk of metabolic acidosis, a common issue in CKD, as plant-based foods generate fewer acid loads compared to animal proteins [126]. The incorporation of more plant-based meals can also reduce the intake of phosphorus and potassium, helping to manage these electrolytes more effectively in CKD patients [127]. Thus, integrating these dietary approaches can be a powerful adjunct to other therapeutic strategies in managing CKD and preventing its progression to more advanced stages [128].

Physical activity, particularly aerobic exercise, positively influences blood pressure control and cardio-renal outcomes in both healthy and CKD patients. Current guidelines recommend 150 min of moderate-intensity aerobic exercise per week or 75 min of vigorous activity spread over three sessions [129,130]. Daily physical exercise is more effective than sporadic exercise in improving vascular health and maintaining better blood pressure levels. Evidence of lifestyle interventions on CKD progression has emerged only in recent years. In a recent prospective study [131], which included 300,000 adults from the UK Biobank, demonstrated that unhealthy behaviors contribute to the transition from a healthy state to First Cardio-Renal-Metabolic Disease (FCRMD), then to Cardiovascular Renal-Metabolic Multimorbidity (CRMM), and ultimately death. The study found that individuals with healthier lifestyles had a lower incidence of CRMM and a slower progression of CKD, highlighting the protective role of one’s lifestyle in delaying disease onset and progression [131]. Diet and exercise improve insulin sensitivity, promoting euglycemia and reducing the risk of microvascular complications [132,133,134]. Hypertension, the leading risk factor for CKD, affects 1 in 7 individuals. Thus, interventions targeting blood pressure through both lifestyle and pharmacological measures significantly delay CKD progression [135,136].

### 4.2. Pharmacological Management

The global burden of CKD management is vast, consuming substantial healthcare resources. Controlling blood pressure is central to slowing CKD progression, and renin–angiotensin system (RAS) inhibitors, including angiotensin-converting enzyme inhibitors (ACEIs) and angiotensin II receptor blockers (ARBs), are foundational in this effort. Both ACEIs and ARBs effectively reduce blood pressure and protect renal function, especially in hypertensive CKD patients. ACEIs, in particular, prevent progression to ESRD by enhancing glomerular selectivity and reducing protein filtration [137]. This effect is most pronounced in patients with significant proteinuria. ACEIs reduce the risk of cardiovascular events (odds ratio [OR] 0.73, 95% CI 0.64–0.84); cardiovascular mortality (OR 0.73, 95% CI 0.63–0.86); and all-cause mortality (OR 0.77, 95% CI 0.66–0.91) compared to placebos [138]. Patients who discontinued ACEIs saw worsening blood pressure and kidney function, despite other medications like calcium channel blockers or β-blockers [139]. Although ACEIs can cause hyperkalemia and coughing, they remain superior to ARBs, particularly in preventing cardio-renal events [138]. Managing anemia and correcting mineral metabolism disturbances are critical components in reducing cardiovascular risk and slowing CKD progression [139]. Anemia, often a consequence of erythropoietin deficiency and iron deficiency in CKD patients, can be effectively treated with erythropoiesis-stimulating agents (ESAs) and iron supplementation [140]. Intravenous iron, particularly ferric carboxymaltose or iron sucrose, has demonstrated improvements in both hemoglobin levels and cardiovascular outcomes by reducing LV hypertrophy and enhancing exercise tolerance [141]. Addressing mineral metabolism disturbances, such as hyperphosphatemia and secondary hyperparathyroidism, through the use of phosphate binders, calcimimetics, and vitamin D analogs helps reduce vascular calcification and maintain bone-mineral homeostasis [142].

Sodium–glucose cotransporter 2 inhibitors (SGLT2is), commonly known as gliflozins, are another key therapy in CKD management, particularly for patients with type 2 diabetes (T2DM) and heart failure (HF) and/or coronary artery disease [112,143,144,145]. SGLT2 is mainly expressed in the proximal tubule and regulates glucose reabsorption in the kidneys. By inhibiting SGLT2, these drugs reduce blood glucose levels, enhance glycosuria and natriuresis, and ultimately improve cardiovascular and renal outcomes. SGLT2i therapy results in an initial decrease in eGFR, followed by stabilization and reduced albuminuria [146]. This nephroprotective effect is further enhanced by their antihypertensive properties [146]. Numerous clinical trials, including EMPAREG-OUTCOME, CANVAS, and DECLARE-TIMI58, have demonstrated that SGLT2 inhibitors reduce major cardiovascular and renal endpoints, particularly hospitalizations and mortality due to HF. These benefits extend even to advanced CKD patients, where SGLT2i therapy slows eGFR decline and improves renal outcomes [147,148,149].

Dyslipidemia is common in CKD, often presenting as elevated low-density lipoprotein cholesterol (LDL-C) and triglycerides in patients with nephrotic-range proteinuria [150]. However, HDL levels are often normal in nephrotic patients [151]. CKD patients can present with different patterns of dyslipidemia, suggesting a multifactorial genesis, including factors like insulin resistance, inflammation, and secondary hyperparathyroidism [150,152,153]. High renal cholesterol levels contribute to chronic inflammation and glomerular sclerosis, supporting the development of interstitial fibrosis and accelerating CKD progression, even in patients without previous nephropathy [154,155]. The altered lipid profile in nephrotic syndrome results from endothelial dysfunction, the increased hepatic synthesis of lipoproteins, and impaired lipoprotein lipase activity due to the elevated oncotic pressure secondary to proteinuria [156]. Statins, which inhibit the HMG-CoA reductase, are widely used to treat dyslipidemia and reduce cardiovascular risk, lowering LDL-C by over 40% in nephrotic patients and potentially slowing progression to ESRD [157]. These drugs have been documented to reduce total and LDL cholesterol significantly and also display an anti-proteinuric effect, contributing to delayed progression to terminal uremia [158]. Although CKD is not considered equivalent to coronary heart disease, its presence correlates with increased CVD incidence compared to patients without CKD [159,160,161]. Meta-analyses including randomized control trials such as AURORA and SHARP, suggest that CKD patients with high cardiovascular risk should target an LDL-C level of 1.8 mmol/L through statins or ezetimibe, or a combination of both [150,162].

Iron deficiency is also prevalent in CKD patients due to chronic inflammation, impaired erythropoietin production, and elevated circulating hepcidin levels, which worsen serum iron levels as eGFR decreases [163]. Intravenous iron supplements, such as ferric carboxymaltose and iron sucrose, improve symptoms in patients with heart failure and anemia and potentially slow CKD progression by enhancing renal function [164]. Therapy with IV ferric carboxymaltose improves heart failure symptoms, anemia, and physical activity resistance, while IV iron sucrose is associated with improved systolic and diastolic function [164]. Lastly, CKD patients receiving iron supplementation, either orally or intravenously, may experience a slower disease progression, underscoring the utility of iron therapy in this population [165].

### 4.3. Dialysis and Cardiovascular Risk

CVD is the leading cause of death in patients with ESRD undergoing hemodialysis (HD), with mortality rates that are up to 20 times higher than in the general population. This increased risk is primarily due to LV hypertrophy and chronic volume overload, compounded by anemia, inflammation, and oxidative stress [159]. A 2019 meta-analysis explored cardiovascular outcomes in HD patients, noting high rates of cardiovascular events, particularly among those with pre-existing cardiovascular risk factors [160]. The findings suggest a need for further research into subpopulations of ESRD patients who are hyporesponsive to erythropoiesis-stimulating agents, as these individuals show higher rates of MACE, even in the absence of significant correlations between ESA therapy and reduced cardiovascular risk [160].

Alternative dialysis methods and emerging technologies offer potential strategies to mitigate cardiovascular risk in patients with ESRD [166]. Peritoneal dialysis (PD), for example, has been associated with the better preservation of residual renal function and a lower incidence of LV hypertrophy compared to traditional hemodialysis, potentially reducing cardiovascular strain [167,168]. Although PD may not be suitable for all patients, studies suggest that it is a viable option for reducing cardiovascular mortality, especially in the early stages of dialysis initiation [167,169]. Additionally, home hemodialysis (HHD), particularly with more frequent or nocturnal sessions, has been shown to provide better fluid control, lower blood pressure, and reduce the LV mass [170]. These improvements translate into a lower risk of cardiovascular events compared to conventional in-center HD schedules [171].

Emerging technologies are also being explored to enhance cardiovascular outcomes in dialysis patients [172]. The development of bioartificial kidneys and wearable dialysis devices aims to provide more continuous and physiological ultrafiltration, which may help prevent the cardiovascular complications associated with intermittent fluid overload [173,174,175]. Moreover, advances in dialysis membranes with improved biocompatibility are being designed to reduce inflammation and oxidative stress, factors closely linked to cardiovascular morbidity in this population [176]. These alternative methods and innovations represent promising avenues for reducing cardiovascular risk and improving overall outcomes in ESRD patients on dialysis.

## 5. Special Considerations and Future Directions

### 5.1. Emerging Therapeutic Approaches for Cardiovascular Complications in CKD

As previously highlighted, the complex interplay between CKD and CVD is driven by multiple factors, including hypertension, hypervolemia, oxidative stress, inflammation, and the dysregulation of RAAS [177]. Recent advances in pharmacotherapy and clinical research provide new hope for addressing these challenges. Among the emerging treatments for CKD and its associated cardiovascular complications, endothelin receptor antagonists have garnered attention for their ability to address key pathological processes. Elevated levels of endothelin-1, a potent vasoconstrictor, contribute to vasoconstriction, fibrosis, and inflammation, all of which exacerbate cardiovascular risk in CKD patients [178]. Atrasentan, a selective endothelin A receptor antagonist, has shown significant potential in this regard. In the SONAR trial, atrasentan was found to slow the progression of CKD, particularly in patients with diabetic nephropathy. By reducing cardiovascular risk and preserving renal function, this therapy offers hope for delaying or even preventing the progression to ESRD in vulnerable populations [179]. Another promising therapeutic option is finerenone, a novel non-steroidal mineralocorticoid receptor antagonist (MRA) [180]. Traditional steroidal MRAs, such as spironolactone, though effective, come with significant risks, including hyperkalemia and gynecomastia. Finerenone, however, offers a more selective approach, minimizing these adverse effects while maintaining therapeutic efficacy. This distinction has been particularly beneficial for CKD patients with concomitant type 2 diabetes mellitus (T2DM). Both the FIDELIO-DKD and FIGARO-DKD trials demonstrated finerenone’s ability to slow CKD progression and reduce cardiovascular complications, offering a safer alternative to traditional therapies for managing CKD in this high-risk population [180,181].

### 5.2. Key Ongoing Clinical Trials

Several ongoing clinical trials are exploring innovative therapies for reducing cardiovascular complications in CKD patients:

FIND-CKD (NCT05047263): this randomized, double-blind, phase 3 trial investigates finerenone’s efficacy and safety in non-diabetic CKD patients, compared to a placebo, alongside standard care [182].

EMPA-KIDNEY (NCT03594110): following the success of previous SGLT2 inhibitor trials, the EMPA-KIDNEY trial has shown that empagliflozin significantly reduces the risk of CKD progression and cardiovascular mortality [183].

FLOW (NCT03819153): this trial evaluates semaglutide’s effect on kidney disease progression and cardiovascular outcomes in CKD patients [184].

SELECT (NCT03574597): the SELECT trial has revealed that semaglutide reduces the risk of MACE by 20% in patients with cardiovascular disease and obesity, even in the absence of diabetes [185,186].

### 5.3. Potential Future Treatments

Gene therapy holds immense promise in addressing the genetic causes of CKD and its cardiovascular complications. Among the most promising techniques is CRISPR-based gene editing, which allows for precise modifications of genes associated with diseases. In fact, CRISPR technology enhances gene editing efficiency by guiding Cas proteins to a precise location in the genome. This is achieved by modifying the base sequence of a small segment of guide RNA, broadening the potential applications of gene-editing technology [187]. In the case of autosomal dominant polycystic kidney disease (ADPKD), CRISPR was used to delete the *PKD2* gene, facilitating a deeper understanding of the disease’s pathogenesis [188]. Additionally, a recent study explored the potential of acutely blocking *Pkd1* and *Pkd2* cis-inhibition using anti-miR-17 oligonucleotides such as RGLS4326. This compound has been shown to increase *Pkd1*/*Pkd2* expression and reduce cyst growth in CRISPR-edited cellular and mouse models of ADPKD. When treated with RGLS4326, cells exhibit reduced proliferation, smaller cyst sizes, and a decreased expression of key proteins involved in disease progression, including Yap1 and c-Myc. This suggests that the acute derepression of *Pkd1*/*Pkd2* may prevent disease onset or even halt cyst growth in established PKD [188]. This groundbreaking technology has the potential to revolutionize treatment, not only by correcting genetic mutations that contribute to CKD but also by offering new avenues for cell therapy and organ transplantation. The ability to modify specific disease-related genes through CRISPR may provide long-lasting solutions to conditions that were previously untreatable [189]. In addition to gene therapy, anti-inflammatory strategies are becoming a focal point in managing the cardiovascular burden of CKD. Chronic inflammation is a key driver of cardiovascular risk in these patients, exacerbating the progression of both CKD and CVD. Emerging therapies, such as monoclonal antibodies that target pro-inflammatory cytokines, are being actively investigated. For example, canakinumab, an IL-1β inhibitor, has shown potential in reducing cardiovascular events by dampening inflammatory responses, a promising strategy for improving long-term outcomes in CKD patients [190,191]. Recent evidence from the RESCUE trial has further highlighted the potential of anti-inflammatory approaches, as ziltivekimab, an IL-6-targeting antibody, significantly reduced biomarkers of inflammation and thrombosis in CKD patients with elevated CRP levels [192]. Furthermore, established anti-diabetic therapies, such as SGLT2i and glucagon-like peptide 1 receptor agonists, have demonstrated kidney and cardiovascular protection that extends beyond glucose control, likely due to their anti-inflammatory effects [41,193]. Another promising agent is finerenone, a non-steroidal selective mineralocorticoid receptor antagonist, which not only reduces CKD progression and cardiovascular events in patients with diabetic CKD but also demonstrates anti-inflammatory potential [194,195].

Genetic and molecular profiling strategies are increasingly becoming integral to the personalized management of CKD, particularly in predicting cardiovascular risk [194]. Advances in next-generation sequencing (NGS) and genome-wide association studies (GWAS) have identified key genetic variants that predispose individuals to CKD and its complications [195]. For example, variations in the UMOD gene, which encodes uromodulin, have been linked to the increased risk of CKD and cardiovascular events [196]. Molecular profiling techniques, such as single-cell RNA sequencing, are now being applied to dissect the specific cellular pathways involved in CKD progression [197]. These technologies enable the precise identification of molecular targets for therapy, as seen in ongoing trials targeting APOL1 risk variants in African American populations with CKD [198,199].

Epigenetic modifications, including DNA methylation and histone acetylation, are also under investigation as potential biomarkers for CKD and cardiovascular disease [200]. Profiling patients for these molecular changes may allow for earlier detection and intervention, particularly in subpopulations with a high risk of rapid disease progression [201]. For instance, the identification of specific microRNAs involved in renal fibrosis, such as miR-21, has opened the door for therapeutic strategies aimed at modulating these non-coding RNAs to slow CKD progression and reduce cardiovascular risk [202].

Oxidative stress, particularly at the level of mitochondrial dysfunction, plays a critical role in both CKD progression and the development of CVD. As a result, researchers are exploring novel antioxidants designed to specifically target mitochondrial damage. These include inhibitors of mitochondrial fission and activators of sirtuins, a family of NAD-dependent deacetylases involved in regulating mitochondrial metabolism. By addressing mitochondrial dysfunction, these therapies aim to slow the progression of CKD while simultaneously reducing cardiovascular risk [203,204]. Early studies suggest that targeting oxidative stress through these mechanisms could represent a major breakthrough in CKD management [205,206,207].

Stem cell therapy and regenerative medicine also offer exciting possibilities for treating CKD and its cardiovascular complications. Mesenchymal stem cells (MSCs), which have shown promise in early-stage trials, are being investigated for their ability to repair damaged kidney tissues in patients with diabetic and hypertensive nephropathy. Beyond their regenerative potential, MSCs are being explored as delivery systems for nephroprotective proteins, such as Klotho. Originally identified as an anti-aging gene, Klotho exerts multiple protective effects, including reducing inflammation, oxidative stress, and fibrosis, while also modulating RAAS. This multifaceted protein holds potential for mitigating both renal and cardiovascular damage in CKD patients [208,209,210]. Emerging therapeutic approaches are summarized in Table 1.

### 5.4. Multifactorial Approach and Challenges in Drug Availability Due to Impaired Renal Function

Managing cardiovascular complications in CKD requires a multifactorial approach that integrates various therapeutic strategies targeting blood pressure, dyslipidemia, albuminuria, anemia, and chronic inflammation [211,212,213]. However, the impaired renal function characteristic of CKD often complicates drug selection and dosing, limiting the availability and safety of certain pharmacological options [214,215,216]. Many standard cardiovascular medications, including RAAS inhibitors, statins, and antidiabetic drugs, must be adjusted or avoided due to altered pharmacokinetics and the risk of adverse effects such as hyperkalemia, nephrotoxicity, or drug accumulation [217,218]. Additionally, newer agents, though promising, may face restrictions in use among CKD patients with advanced disease stages due to concerns about renal clearance and side effects [219,220,221]. This highlights the ongoing need for personalized medical approaches, careful monitoring, and the development of renal-safe formulations to optimize therapeutic outcomes while minimizing harm in this vulnerable population [194,222,223]. Ongoing research into genetic and molecular profiling will enable clinicians to tailor treatment plans to each patient’s unique risk factors, offering more precise interventions that target the root causes of CKD and CVD [224,225,226,227,228].

### 5.5. Research Gaps

To advance the understanding and treatment of CKD and its cardiovascular complications, it is essential to address several open research gaps. First, the long-term efficacy and safety of CRISPR-based gene editing techniques in vivo remain largely uncharted, necessitating studies that evaluate the durability of gene corrections and potential off-target effects [227]. Additionally, the specific mechanisms linking chronic inflammation to cardiovascular risk in CKD require further investigation to inform targeted therapeutic approaches [228]. The integration of genetic and molecular profiling into clinical practice is another critical area, as standardized protocols could enhance personalized treatment strategies [224]. Moreover, understanding the role of epigenetic modifications and oxidative stress mechanisms in CKD progression presents opportunities for novel intervention strategies [229]. The efficacy of stem cell therapies and their underlying mechanisms needs validation through large-scale trials, while the interplay between CKD and comorbid conditions like diabetes must be elucidated to inform integrated treatment approaches [230]. Lastly, establishing causal relationships in biomarker discovery is crucial for determining their predictive value and therapeutic potential [231,232]. Addressing these gaps will pave the way for innovative strategies that improve outcomes for CKD patients and mitigate their cardiovascular risk.

## 6. Conclusions

In conclusion, CKD remains a significant public health issue that requires multifaceted approaches for prevention and management. By addressing the underlying risk factors, implementing effective therapeutic strategies, and advancing research into emerging treatments, we can significantly improve the quality of life and outcomes for patients with CKD. Future studies should focus on personalized medical approaches to tailor interventions for high-risk populations, ensuring optimal care and resource allocation.

## Figures and Tables

**Table 1:** Emerging therapeutic approaches for cardiovascular complications in CKD.

Medication	Clinical Trial	Population (Number of Patients)	Intervention	Comparison	Outcome	Notable Effects/ Considerations	Ref
**Atrasentan**	SONAR	Diabetic nephropathy, CKD patients (2648)	Endothelin receptor antagonist (atrasentan)	Placebo or standard therapy	Slows CKD progression and reduces vasoconstriction, fibrosis, inflammation, and cardiovascular risk	Particularly beneficial in diabetic nephropathy	[179]
**Finerenone**	FIDELIO-DKD	Diabetic kidney disease patients with CKD (5734)	Non-steroidal mineralocorticoid receptor antagonist	Placebo or steroidal MRAs	Reduced CKD progression and cardiovascular events	Lower risk of hyperkalemia compared to steroidal MRAs	[180]
**Finerenone**	FIGARO-DKD	Diabetic kidney disease patients with earlier-stage CKD (7352)	Non-steroidal mineralocorticoid receptor antagonist	Placebo or steroidal MRAs	Reduced cardiovascular events and slower CKD progression	Beneficial even in patients with early CKD	[181]
**Empagliflozin**	EMPA-KIDNEY	CKD patients, particularly with type 2 diabetes (6609)	SGLT2 inhibitor	Placebo or standard therapy	Decreases CKD progression and cardiovascular mortality	Provides cardiovascular and renal protection independent of glycemic control	[183]
**Semaglutide**	FLOW	CKD patients with type 2 diabetes (3000)	GLP-1 receptor agonist	Placebo or standard therapy	Slowed CKD progression	Potential to slow kidney disease progression alongside glucose control	[184]
**Semaglutide**	SELECT	Obese patients with cardiovascular disease (17,604)	GLP-1 receptor agonist	Placebo or standard therapy	Reduced risk of MACE by 20% in patients with obesity and cardiovascular disease	Cardiovascular benefits independent of glucose control	[185]
**CRISPR Gene Therapy**	Ongoing research	Patients with genetic causes of CKD (ongoing)	CRISPR-based gene editing	Standard treatment or no gene therapy	Potential for long-lasting correction of genetic mutations; still experimental	Promising but undergoing ongoing research	[177]
**Canakinumab**	CANTOS	CKD patients with increased cardiovascular risk (10,061)	IL-1β inhibitor	Placebo or standard therapy	Reduces cardiovascular events by targeting inflammatory pathways	Anti-inflammatory effects	[191]
**Ziltivekimab**	RESCUE	CKD patients with high CRP levels (623)	IL-6 inhibitor	Placebo	Reduces inflammation and thrombosis	Shows promise in managing inflammation-related CKD complications	[192]
**Mesenchymal Stem Cells (MSCs)**	Early-stage trials	CKD patients, particularly with diabetic or hypertensive nephropathy (ongoing)	Stem cell therapy	Standard therapy or no MSC therapy	Repairs damaged kidney tissue and reduces fibrosis and inflammation; early-stage trials	Promising for nephroprotection and delivery of Klotho	[208,209]

## Data Availability

No dataset was generated for the publication of this article.
